# Current evidence on the impact of medication optimization or pharmacological interventions on frailty or aspects of frailty: a systematic review of randomized controlled trials

**DOI:** 10.1007/s00228-020-02951-8

**Published:** 2020-08-07

**Authors:** Farhad Pazan, Mirko Petrovic, Antonio Cherubini, Graziano Onder, Alfonso J. Cruz-Jentoft, Michael Denkinger, Tischa J. M. van der Cammen, Jennifer M. Stevenson, Kinda Ibrahim, Chakravarthi Rajkumar, Marit Stordal Bakken, Jean-Pierre Baeyens, Peter Crome, Thomas Frühwald, Paul Gallaghar, Adalsteinn Guðmundsson, Wilma Knol, Denis O’Mahony, Alberto Pilotto, Elina Rönnemaa, José Antonio Serra-Rexach, George Soulis, Rob J. van Marum, Gijsbertus Ziere, Alpana Mair, Heinrich Burkhardt, Agnieszka Neumann-Podczaska, Katarzyna Wieczorowska-Tobis, Marilia Andreia Fernandes, Heidi Gruner, Dhayana Dallmeier, Jean-Baptiste Beuscart, Nathalie van der Velde, Martin Wehling

**Affiliations:** 1 Clinical Pharmacology Mannheim, Faculty of Medicine Mannheim, Ruprecht-Karls-University of Heidelberg, Theodor-Kutzer-Ufer 1-3, 68167 Mannheim, Germany; 2 Section of Geriatrics, Department of Internal Medicine and Paediatrics, Ghent University, Ghent, Belgium; 3 Department of Geriatric Medicine, INRCA Istituto Nazionale di Ricovero e Cura per Anziani, Ancona, Italy; 4 Department of Cardiovascular, Endocrine-Metabolic Diseases and Aging, Istituto Superiore di Sanità, Rome, Italy; 5 Servicio de Geriatría. Hospital Universitario Ramón y Cajal (IRYCIS), Madrid, Spain; 6 Department of Geriatrics, University of Ulm and Geriatric Center Ulm/Alb-Donau, Agaplesion Bethesda Hospital, Alb-Donau, Ulm, Germany; 7 Faculty of Industrial Design Engineering, Delft University of Technology, Delft, Netherlands; 8 Institute of Pharmaceutical Science, King’s College London, London, UK; 9 Academic Geriatric Medicine, Faculty of Medicine, Southampton University, Southampton, UK; 10 Geriatric & Stroke Medicine, Brighton and Sussex Medical School, Brighton, UK; 11 Department of Clinical Science, University of Bergen, Bergen, Norway; 12 AZ Alma, Eeklo, Belgium; 13 University of Luxembourg, Esch-sur-Alzette, Luxembourg; 14 Keele University, Newcastle under Lyme, UK; 15 Department of Acute Geriatrics, Social Medical Center East, Vienna, Austria; 16 School of Medicine, University College Cork, Cambridge, UK; 17 University of Iceland, Geriatrics K4, Landspítali University, Reykjavík, Iceland; 18 Geriatrics, Department of Geriatric Medicine and Expertise Centre Pharmacotherapy in Old Persons, University Medical Center Utrecht, Utrecht University, Utrecht, The Netherlands; 19 Department of Medicine, University College Cork, Cork University Hospital, Cork, Ireland; 20 Department of Geriatric Care, Orthogeriatrics and Rehabilitation, Galliera Hospital, Genoa, Italy; 21 Department of Interdisciplinary Medicine, University of Bari, Bari, Italy; 22 Department of Public Health and Caring Sciences/Geriatrics, Uppsala, Sweden; 23 Geriatric Department, Hospital General Universitario Gregorio Marañón, Madrid, Spain; 24 Instituto de Investigación Sanitaria Gregorio Marañón, Madrid, Spain; 25 Consorcio de Investigación Biomédica en Red: Fragilidad y Envejecimiento Saludable, CIBERFES, Madrid, Spain; 26 Medicine Department, Facultad de Medicina, Universidad Complutense de Madrid, Madrid, Spain; 27 Outpatient Geriatric Assessment Unit, Henry Dunant Hospital Center, Athens, Greece; 28 Department of General Medicine and Geriatric Medicine, Free University, Amsterdam, The Netherlands; 29 Section of Geriatric Medicine, Department of Internal Medicine, Erasmus MC, University Medical Center Rotterdam, Rotterdam, The Netherlands; 30 Department of Geriatric Medicine, Maasstad Hospital, Rotterdam, The Netherlands; 31 Effective Prescribing and Therapeutics, Scottish Government, Edinburgh, EH1 3DG UK; 32 IV. Medical Department, Geriatrics, University Hospital Mannheim, Heidelberg University, Mannheim, Germany; 33 Geriatric Laboratory Palliative Medicine Department, Poznan University of Medical Sciences, Poznan, Poland; 34 Department of Palliative Medicine, Poznan University of Medical Sciences, Poznan, Poland; 35 Palliative Medicine Unit, University Hospital of Lord’s Transfiguration, Poznan, Poland; 36 Department of Internal Medicine, Hospital Curry Cabral, Centro Hospitalar Universitário de Lisboa Central, Lisbon, Portugal; 37 Internal Medicine Department 7.2, Hospital Curry Cabral - Centro Hospitalar Universitário Lisboa Central, EPE, Lisbon, Portugal; 38 AGAPLESION Bethesda Clinic Ulm, Ulm, Germany; 39 Dept. of Epidemiology, Boston University School of Public Health, Boston, MA USA; 40 CHU Lille, ULR 2694 - METRICS: Évaluation des technologies de santé et des pratiques médicales, Univ. Lille, F-59000 Lille, France; 41 Department of Geriatric Medicine, Free University, Amsterdam, the Netherlands

**Keywords:** Frailty, Prefrailty, Polypharmacy, Medication optimization, Inappropriate drug treatment, Older people

## Abstract

**Background:**

Frailty and adverse drug effects are linked in the fact that polypharmacy is correlated with the severity of frailty; however, a causal relation has not been proven in older people with clinically manifest frailty.

**Methods:**

A literature search was performed in Medline to detect prospective randomized controlled trials (RCTs) testing the effects of pharmacological interventions or medication optimization in older frail adults on comprehensive frailty scores or partial aspects of frailty that were published from January 1998 to October 2019.

**Results:**

Twenty-five studies were identified, 4 on comprehensive frailty scores and 21 on aspects of frailty. Two trials on comprehensive frailty scores showed positive results on frailty although the contribution of medication review in a multidimensional approach was unclear. In the studies on aspects related to frailty, ten individual drug interventions showed improvement in physical performance, muscle strength or body composition utilizing alfacalcidol, teriparatide, piroxicam, testosterone, recombinant human chorionic gonadotropin, or capromorelin. There were no studies examining negative effects of drugs on frailty.

**Conclusion:**

So far, data on a causal relationship between drugs and frailty are inconclusive or related to single-drug interventions on partial aspects of frailty. There is a clear need for RCTs on this topic that should be based on a comprehensive, internationally consistent and thus reproducible concept of frailty assessment.

**Electronic supplementary material:**

The online version of this article (10.1007/s00228-020-02951-8) contains supplementary material, which is available to authorized users.

## Introduction

Frailty has been defined by the World Health Organization as a ‘progressive age-related decline in physiological systems that results in decreased reserves of intrinsic capacity, which confers extreme vulnerability to stressors and increases the risk of a range of adverse health outcomes’ [1, 2]. While there is still some debate concerning a more precise definition of frailty, this widely recognized definition of frailty was also implemented by the first joint action (JA) on the prevention of frailty, ADVANTAGE [1, 2]. The prevalence of frailty increases with age (about 11% in community-dwelling older adults [3–6]); frailty is a dynamic process and older people are commonly staged as being robust/non-frail, prefrail, or frail [3, 5]. The presence of frailty in older adults has been associated with serious adverse clinical outcomes including, falls, disability, hospitalization, nursing home admissions, and even mortality [3, 4, 7].

There are many instruments designed to define and measure frailty or different aspects of frailty including its physical, cognitive, social, environmental, and emotional domains [8, 9]. The most commonly used instruments are the frailty phenotype and the Frailty Index [10]. However, according to the ADVANTAGE JA initiative, validated instruments such as the Short Physical Performance Battery (SPPB), the Edmonton Frail Scale, (The Rockwood) Clinical frailty Scale, or PRISMA-7 which do not require special equipment and take less than 10 min to complete are useful for frailty screening and preferable to others across clinical settings [1, 2].

In a previous publication examining the association of polypharmacy (i.e., often defined as ≥ 5 daily medications) and hyperpolypharmacy (i.e., ≥ 10 daily medications) with frailty, we found that polypharmacy is common in prefrail and frail adults and that robust/non-frail persons with polypharmacy are at significantly higher odds for developing prefrailty compared to those not exposed to polypharmacy [5]. This association between polypharmacy, inappropriate prescribing, and frailty has been corroborated by several systematic reviews that suggest, but do not prove, that a reduction of polypharmacy may prevent or improve frailty or aspects of frailty [11–13]. In this context, an important limitation of interpreting evidence from observational studies on medications and frailty has to be noted: clinicians often selectively discontinue/de-prescribe in those who are frailest, thereby creating a bias regarding applicability to frail people at large. As these associations do not provide insights on the causal relationship between medications and frailty, more research is required to address this interaction that could reflect medications being increased to cope with frailty or frailty as a consequence of medications. A lack of evidence on pharmacological interventions for the management of frailty has been anticipated, and already been indicated in literature [14–16]. Therefore, a systematic review was performed to identify evidence on pharmacological interventions and frailty or aspects of frailty from randomized controlled trials (RCT). Approaches to tackle polypharmacy and inappropriate drug treatment (medication optimization), as well as single-drug interventions (pharmacological interventions) aimed at improving clinically manifest frailty as a primary or secondary outcome, were searched for by addressing both key aspects of medication-frailty interactions: aggravating or improving frailty through medication.

## Methods

This systematic review was performed according to the methodological manuals of the Preferred Reporting Items for Systematic Reviews and Meta-Analyses (PRISMA [17]). The PRISMA checklist and PICOs are provided in Supplementary data 1 & 2. It was an initiative of the European Geriatric Medicine Society (EuGMS) special interest group (SIG) on Pharmacology.

### Search strategy

Search terms were proposed by two authors (FP and MW) to all EuGMS Pharmacology special interest group members for discussion and amendment. The resultant search terms (Supplementary Material 3), combinations, and limitations were developed and used to search MEDLINE, from January 1998 up to and including 14^th^ October 2019. The key themes in our search were frailty and medicines (including polypharmacy and inappropriate prescribing), and only randomized controlled trials (RCT) were included.

### Inclusion criteria

Publications describing the impact of medication optimization or single-drug interventions on frailty or aspects of frailty in older adults (≥ 60 years old, except one study [18]), evaluated through a randomized controlled trial were examined. A broad definition of medication optimization was used including not only medication review, but also educational interventions, care coordination, use of technology, or ‘brown bag’ analyses.

### Exclusion criteria

**Fig. 1:**
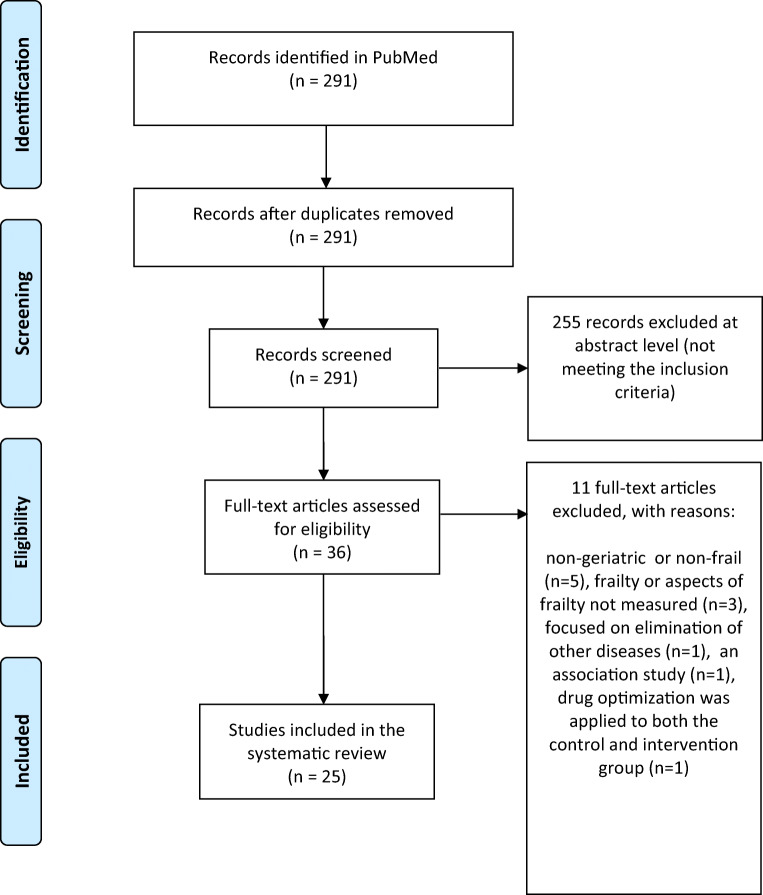
Flow diagram of randomized controlled trials (RCT) on medication optimization or pharmacological interventions in frail older patients and its impact on frailty (PRISMA)

Studies concerning non-geriatric or non-frail (at baseline) patients were excluded, as were studies without measurement of frailty or aspects of frailty, or studies measuring quality of life without providing a separate analysis of the intervention on the aforementioned aspects of frailty. These exclusion criteria also applied to nursing homes, though a considerable rate of frail patients is to be expected there, but the measurement of interventional improvements is not possible without baseline data on frailty. Studies focusing on treatment of diseases such as most oncology trials, association studies, or studies without differences in medications between the control and intervention group were also excluded (Fig. 1). There were no exclusions regarding the language.

### Study selection

The search results were exported by FP from PubMed to a Word file (Microsoft, Redmond, Washington). Subsequently, two reviewers (FP, MW) independently screened the titles and abstracts of the manuscripts to identify relevant publications describing the impact of medication optimization or drug treatment on frailty or aspects of frailty in randomized controlled trials. Each record generating uncertainty regarding inclusion or exclusion criteria was discussed by FP and MW in order to reach consensus about inclusion.

### Data extraction and synthesis

The following data were extracted from the selected publications: PubMed ID (PMID), first author, publication year, type of population, mean age of study participants and standard deviation if provided, number of study participants, female gender, outcome relating to a common frailty score/instrument, outcome(s) relating to partial aspects of frailty, short description of the intervention and its duration, positive outcome(s) relating to frailty or aspects of frailty. Methodological quality, or risk of bias of clinical trials, was calculated by using a three-item questionnaire, known as the Jadad score [19]. Drop-outs/withdrawals, randomization, blinding, and the quality of latter two items are assessed and a score derived ranging from 0 (very poor) to 5 (rigorous) [19]. In the assessment of the trials, positive study outcomes corresponded to at least one primary or secondary endpoint exposing a significant improvement by the intervention (i.e., *p* < 0.05).

### Measurement of frailty or aspects of frailty considered for study selection and data extraction

Measurement of frailty corresponded to highly cited [20] or other commonly used/recommended (by ADVANTAGE JA) frailty instruments [5, 14]. The following tools were considered to be comprehensive frailty scores: (physical) frailty phenotype (PFP, also known as Fried Frailty Criteria), Deficit Accumulation Index (DAI), Frailty index, Electronic Frailty Index, Gill Frailty Measure, Frailty/Vigor assessment, (The Rockwood) Clinical Frailty Scale, Brief Frailty Instrument, Vulnerable Elders Survey (VES-13), Fatigue, Resistance, Ambulation Illness, Loss of Weight Index (FRAIL Index), Inter-Frail, Sherbrooke Postal Questionnaire, Groningen Frailty Indicator, Study of Osteoporotic Fractures frailty criteria, Tilburg Frailty Indicator, Edmonton Frailty Scale, Frail Scale, Short Physical Performance Battery (SPPB), PRISMA-7, Multidimensional Prognostic Index, Geriatric 8 frailty questionnaire for oncology (G8), Kihon Checklist, Frailty Risk score, Hospital Frailty Risk Score, and Winograd Screening Instrument.

The aspects of frailty considered to be relevant included physical performance/function, body composition, body weight/weight loss, cognition, exhaustion/fatigue, and muscle strength. We particularly focused on the following assessments: gait speed, walking speed, activities of daily living (ADL), instrumental activities of daily living (IADL), Timed Up and Go test (TUG), handgrip strength, and Mini Mental State Examination (MMSE).

## Results

### Study selection

The search yielded 291 studies, of which 255 were excluded at the abstract level (Fig. 1**)**. The remaining 36 studies were reviewed in full-text and 11 were excluded based on the abovementioned criteria from this systematic review, leading to the selection of 25 articles [18, 21–44]. Finally, only 4 of the 25 studies measured the frailty status by a ‘comprehensive’ frailty instrument as an endpoint, and the remaining 21 studies detected changes in partial aspect(s) of frailty as an outcome (Table 1). The total number of study participants, types of intervention, number of trials with positive outcome(s), and the number of trials with a Jadad score ≥ 3 (a trial with a score above 2 is considered to have a high quality [45]) are provided in Table 1.

**Table 1:** Results of the structured comprehensive review on interventional medication optimization or pharmacological intervention and its impact on frailty and/or partial aspects of frailty

	Total number of trials	Number of study participants	Number of trials on single-drug intervention/number of trials with medication optimization	Number of singular intervention trials/number of multi-interventional trials	Number of trials with positive outcome(s) related to a comprehensive frailty score^a^	Number of trials with positive outcome(s) related to partial aspects of frailty^b^	Number of studies with a Jadad score^c^ of 3 or over
Interventional trials with a comprehensive frailty score as one endpoint	4	1147	1 3	1 3	2	3	2
Interventional trials with partial aspects of frailty as one endpoint	21	3807	13 8	15 6	–	13	13

^a^The following tools were considered to be comprehensive frailty scores: (Physical) Frailty Phenotype (PFP, also known as Fried Frailty Criteria), Deficit Accumulation Index (DAI), Frailty index, Electronic Frailty Index, Gill Frailty Measure, Frailty/Vigor assessment, Clinical Frailty Scale, Brief Frailty Instrument, Vulnerable Elders Survey (VES-13), Fatigue, Resistance, Ambulation Illness, Loss of Weight Index (FRAIL Index), Inter-Frail, Sherbrooke Postal Questionnaire, Groningen Frailty Indicator, Study of Osteoporotic Fractures frailty criteria, Tilburg Frailty Indicator, Edmonton Frailty Scale, Frail Scale, Short Physical Performance Battery (SPPB), PRISMA-7, Multidimensional Prognostic Index, Geriatric 8 frailty questionnaire for oncology (G8), Kihon Checklist, Frailty Risk score, Hospital Frailty Risk Score and Winograd Screening Instrument

^b^The aspects of frailty considered to be relevant included physical performance/function, body composition, body weight/weight loss, cognition, exhaustion/fatigue, strength, and memory. We particularly focused on the following assessments: gait speed, walking speed, activities of daily living (ADL), instrumental activities of daily living (IADL), Timed Up and Go test (TUG), handgrip strength, and Mini Mental State Examination (MMSE)

^c^The Jadad score which is a scale to assess the methodological quality or risk of bias of clinical trials is calculated by using a three-item questionnaire. Drop-outs/withdrawals, randomization, blinding, and the quality of latter two items are assessed. The derived score ranges from zero (very poor) to five (rigorous). Jadad AR, Moore RA, Carroll D, et al. Assessing the quality of reports of randomized clinical trials: is blinding necessary? Control Clin Trials. 1996 Feb;17:1–12

### Studies including a comprehensive frailty score as an outcome (*n* = 4)

In only two out of the four studies [23, 24] with a comprehensive frailty score, a significant improvement of the frailty status by a multidisciplinary intervention that included medication review was demonstrated. Both studies used the Short Physical Performance Battery (SPPB) as frailty instrument and applied a multidimensional intervention (Supplementary Table 1a & 1b). In the study conducted by Romera-Liebana et al. [23] which recruited frail older persons living in the community, a 12-week intervention was utilized consisting of exercise training, intake of high protein nutritional drinks/supplements, memory training, and medication review. Other aspects of frailty (handgrip strength, Functional Reach Test, Unipodal Station Test, and several neuropsychological performance tests) were measured or performed in addition to the SPPB. In this trial, handgrip strength, Functional Reach Test, and the neuropsychological performance tests improved significantly (*p* < 0.05) in the intervention group as compared to the control group. However, this study had a Jadad score of 2, suggesting poor quality. The other study conducted by Matchar et al. [24] recruited participants who visited an emergency department for a fall-related injury and were discharged home. A tailored program of physical therapy for 3 months plus screening and follow-up for vision, polypharmacy, and environmental hazards for 6 months was employed. With a Jadad score of 3, the trial was of better quality than the Romera-Liebana study [23]. SPPB was a secondary outcome which significantly more deteriorated in the control group as compared to the intervention group.

The remaining two studies [21, 22] both used the frailty phenotype (Fried Frailty Criteria) to measure frailty; one study also used SPPB. The study which used SPPB in addition to the frailty phenotype was a single-drug intervention in males (testosterone versus placebo) [21]; the other trial utilized a multidimensional intervention consisting of comprehensive geriatric assessment and appropriate intervention by medication adjustment, exercise instruction, nutrition support, physical rehabilitation, social worker consultation, and specialty referral in community-dwelling older persons. These studies showed no improvement of the comprehensive frailty score. A significant increase in lean body mass by testosterone was observed in one study [21]; this trial had a higher Jadad score as compared to the multi-interventional trial (3 versus 1).

In those 11 studies that included a ‘medication review’ [for categorization see 46], this was a comprehensive prescription review in 1 case, an adherence review in 1 case, a clinical medication review in 7 cases, and others in 2 cases. It was performed by clinicians with different professional backgrounds (multidisciplinary in 6 cases, geriatricians only, GPs only or nurses only in 1 case each) and by physicians and pharmacists or by pharmacists only in 1 case each. Initial access to past medical records was provided in five cases (Supplementary Table 1b).

### Studies including an aspect of frailty as an outcome (*n* = 21)

The majority (*n* = 13) of the trials which only addressed single or multiple aspects of frailty as an endpoint were single-drug interventions [18, 26, 28, 30, 32, 33, 35–40, 42], and in ten of these trials, a significant improvement of some aspects of frailty was demonstrated. Only three trials [32, 38, 39] failed in this regard. Eight trials on single-drug interventions had a Jadad score of 3 or more [26, 28, 29, 32, 35, 36, 39, 40].

In only two of the 21 studies which focused on aspects of frailty, a medication optimization process represented the intervention [27, 28]. These studies showed no impact of the intervention on the aforementioned aspects of frailty.

There were six multi-interventional trials [25, 31, 34, 41, 43, 44] with aspects of frailty as an endpoint; except for [31], they used medication review/optimization as one part of the intervention. Only three of them (including ref. 28) showed a positive impact on some aspects of frailty [25, 34]. However, the Jadad score was below 3 in three of these studies [31, 43, 44].

Supplementary Table 1a & 1b summarizes the 25 randomized trials found in this review and provides relevant details about these studies including type of study population, intervention, the nature of medication review (if applied), outcomes, and quality of the trial according to the Jadad score [19].

An overview of frailty aspects considered in these 25 studies and the frequency and types of interventions with and without significantly positive impact on aspect(s) of frailty are provided in Table 2.

**Table 2:** An overview of frailty aspects in the interventional studies included in this review. The types of interventions with positive effects on at least one aspect of frailty or no impact are described separately

Aspect of frailty	Number of studies using this aspect (thereof one-item interventions)	Number of studies with positive outcome (thereof one-item interventions)	Intervention(s) used in the studies with no impact (separated by a slash)	Intervention(s) used in the studies with positive outcome (separated by a slash)
Physical performance/function^a^ (including TUG, PASE, balance)	17 (12)	9 (6)	Testosterone/spironolactone/MVP regimen/tablets of Chinese herbal formula/recombinant human chorionic gonadotropin/supplementation with a multinutrient liquid supplement/medication review + Falls risk factor assessment + modification and seated balance exercise training program/‘half-day Chronic Care Clinics’. These clinics included an extended visit with the physician and nurse with a special focus on chronic disease management; a pharmacist visit that aimed at a reduction of polypharmacy and high-risk medications; and a patient self-management or support group	Exercise training, intake of high protein nutritional shakes, memory training, and medication review/medication review and optimization of medication use, improvement of physical fitness, social skills and nutrition/alfacalcidol/early switch to oral treatment with diuretics/teriparatide/coordinated care by nurses for two intervention groups who also received either an ‘MD.2 medication-dispensing machine’ or a medplanner (simple box with separate compartments for individual medication times)/piroxicam/testosterone/orally active GHS capromorelin
Strength (including handgrip)	10 (7)	4 (2)	Testosterone/recombinant human chorionic gonadotropin/piroxicam/tablets of Chinese herbal formula/supplementation with multinutrient liquid/medication review, Falls risk factor assessment, modification and seated balance exercise training program	Four component intervention: exercise training, intake of high protein nutritional shakes, memory training, and medication review/medication review and optimization of medication use, improvement of physical fitness, social skills and nutrition/alfacalcidol/testosterone
Body composition and body weight	7 (7)	6 (6)	Piroxicam	Testosterone in 3 studies/orally active GHS capromorelin/s.c. recombinant human chorionic gonadotropin/supplementation with a multinutrient liquid supplement
Cognition, behavioral disturbances and depression (including MMSE)	13 (6)	2 (0)	Medication review and optimization of medication use, improvement of physical fitness, social skills and nutrition/single Multidisciplinary Multistep Medication Review (3MR)/early switch to oral treatment with diuretics/deprescribing intervention, the planned cessation of non-beneficial medicines/spironolactone/levodopa medication withdrawal/MVP regimen/ tablets of Chinese herbal formula/assessment by a nurse on 12 dimensions including drug treatment and recommendations to participants GPs. Monthly telephone calls were made by the nurse to verify if the recommendations had been implemented/medication review, Falls risk factor assessment, modification and seated balance exercise training program/‘half-day Chronic Care Clinics’. These clinics included an extended visit with the physician and nurse with a special focus on chronic disease management; a pharmacist visit that aimed at a reduction of polypharmacy and high-risk medications; and a patient self-management or support group	Exercise training, intake of high protein nutritional shakes, memory training, and medication review/coordinated care by nurses for two intervention groups who also received either an ‘MD.2 medication-dispensing machine’ or a medplanner (simple box with separate compartments for individual medication times)
ADL/IADL	5 (2)	3 (1)	Comprehensive geriatric assessment and appropriate intervention by medication adjustment, exercise instruction, nutrition support, physical rehabilitation, social worker consultation, and specialty referral/MVP regimen	Medication review and optimization of medication use, improvement of physical fitness, social skills and nutrition/early switch to oral treatment with diuretics/high-intensity weight-lifting exercise and treatment of balance, osteoporosis, nutrition, vitamin D + calcium, depression, cognition, vision, home safety, polypharmacy, hip protectors, self-efficacy, and social support
Fatigue	3 (3)	1 (1)	Testosterone/Tablets of a Chinese herbal formula	Piroxicam
Appetite loss	1 (1)	1 (1)	–	MVP regimen
Others (FIM, assistive device utilization, SMAF)	3 (1)	2 (1)	Assessment by a nurse on 12 dimensions including drug treatment and recommendations to participants GPs. Monthly telephone calls were made by the nurse to verify if the recommendations had been implemented	Early switch to oral treatment with diuretics/high-intensity weight-lifting exercise and treatment of balance, osteoporosis, nutrition, vitamin D + calcium, depression, cognition, vision, home safety, polypharmacy, hip protectors, self-efficacy, and social support

*ADL* activities of daily living, *IADL* instrumental activities of daily living, *GHS:* growth hormone secretagogue, *s.c.* subcutaneous, *MVP* mitomycin-C 8 mg/m^2^ d1, vinblastine 4 mg/m^2^ d 1–8, cisplatin 100 mg/m2 d1); Mini-Mental State Examination; *TUG* Timed Up and Go Test, *PASE* Physical Activity Scale for the Elderly, *SPPB* Short Physical Performance Battery, *SMAF* Functional Autonomy Measurement System, *FIM* Functional Independence Measure

^a^Excluding SPPB and frailty phenotype

Physical performance was measured in 17 studies and improved in 9 of them:

- in two studies involving complex interventions,
- in one study involving an early switch to oral treatment with diuretics in patients with heart failure and
- in one study utilizing a home-based support program.

Single-drug interventions with positive impact on physical performance tested

- alfacalcidol (a vitamin D analog),
- teriparatide (an anabolic parathyroid hormone fragment),
- piroxicam (a nonsteroidal anti-inflammatory drug),
- testosterone (an anabolic steroid) or
- capromorelin (a growth hormone secretagogue).

Four of ten interventional trials on muscle strength (including handgrip) showed positive results. In two of these trials, a complex intervention was evaluated; in two other trials, alfacalcidol or testosterone was applied in the intervention group.

Body composition and body weight were positively affected in six of seven interventional trials measuring these parameters. The trials with positive outcomes used single drugs (5)/nutritional supplementation (1) as intervention. The successful single drugs were testosterone in three studies and capromorelin or recombinant human chorionic gonadotropin in one study each.

Cognition, behavioral disturbances, and/or depression were improved in only 2 of 13 trials. One of these trials utilized a complex intervention and the other a home-based support program. Activities of daily living (ADL) and/or instrumental activities of daily living (iADL) improved in three out of five studies. In one of these studies, the early switch to oral diuretics in patients with heart failure positively affected the outcomes and the other two were based on complex interventions. Fatigue was ameliorated by piroxicam, while two other trials with fatigue as an outcome showed no significant improvement. Anorexia was positively influenced by a chemotherapy regimen (MVP: mitomycin-C, vinblastine, cisplatin) as compared to MVC (mitomycin-C, vinblastine, carboplatin) in patients with advanced non-small cell lung cancer.

## Discussion

The present systematic review showed that among the identified 25 studies, only 2 trials on comprehensive frailty scores reported positive results though the contribution of medication review was unclear in a multidimensional approach. In addition, the studies were heterogeneous regarding the nature of the medication review used (Supplementary Table 1b) and, thus, do not allow for identifying the most recommendable type of medication review. In the studies on frailty aspects, ten single-drug interventions were positive for physical performance, muscle strength, or body composition. There were no studies on negative effects of drugs on frailty. To our knowledge, this is the first systematic review addressing the impact of drug interventions on frailty or aspects of frailty.

The association of frailty and drugs has been described repeatedly in the literature, including a recent review by this group [5]. As many medications may cause mental/cognitive and/or physical deterioration, a causal relation between those drugs and major aspects of frailty is plausible. Such side effects or adverse effects constitute the most important reasons to classify them as “potentially inappropriate medications” (PIMs) in PIM lists like the US Beers criteria [47]. Conversely, medications may positively alter aspects of age-related frailty such as acetylcholine esterase inhibitors, angiotensin-converting enzyme (ACE) inhibitors, appetite enhancers, or nutritional supplements/vitamins to increase muscle strength, all of which have been shown to elicit effects principally relevant to frailty. Some of these medications are positively labeled, i.e., to be encouraged in the FORTA (Fit fOR The Aged) list (labels A or B [48]), a positive/negative drug list for age appropriateness of drugs or are recommended in START criteria [49]. It is obvious that frequent medication reviews (every 3–6 months) addressing over- and undertreatment issues are always desirable in multimorbid older patients to avoid noxious side effects and to provide chances of positively labeled drugs, apart from dealing with those aspects of frailty.

It is clearly important to search for evidence to support these bi-directional claims of impact of medication on aspects of frailty. The outcome of this search is, however, very limited as mentioned above. Thus, the impact of medication review in general and of specific medication avoidance in particular on frailty in older people is largely unknown. Randomized controlled trials involving increasing numbers of representative older people using structured medication reviews or individual drug trials as single interventions for frailty are clearly needed. A key driver in this regard could be the European Medicine Agency in that it encourages the use of SPPB or gait speed as instruments in pre- and post-authorization studies for medicine registration across all therapeutic areas [50].

The 21 trials on particular aspects of frailty were more conclusive in that 10 trials with positive outcomes tested single-drug interventions. The medications with positive effects on physical performance, muscle strength, body composition, and fatigue were alfacalcidol, teriparatide, piroxicam, testosterone, recombinant human chorionic gonadotropin, and capromorelin. Notably, none of them proved to be beneficial to improve comprehensive frailty scores. A strong biological understanding of frailty based on basic research is needed before more focused and presumably more successful treatment strategies may be developed.

Many of the trials (*N* = 10) found in this systematic review are of low methodological quality according to the Jadad score, a finding that emphasizes the lack of compelling data and strategies.

It is remarkable that no prospective RCT-derived evidence was found for the potentially harmful effects of medications on frailty symptoms. There is abundant literature on potentially harmful drugs, such as those that predispose to falls, delirium or dementia, drugs that may even increase mortality (e.g., sedative antihistamines) [51, 52]. However, none of them clearly describe the worsening of aspects of frailty or the use of a comprehensive frailty score according to the criteria of this systematic review. For instance, falls are not included in most frailty concepts, but usually considered as an outcome of frailty.

From the findings of the present systematic review, it appears that a classic prospective, controlled RCT designed to examine the harmful effects of medications in age-related frailty would be ethically questionable as it would involve placing already incapacitated patients at potentially higher risk if still exposed to potentially harmful drugs in the control group. Thus, other trial designs (for example, stepped-wedge design) and/or other interventions such as re-prescribing (the replacement of inappropriate medications by better alternatives for the treatment of a given disease, thus reflecting the combined optimization of both over- and undertreatment issues) based on medication reviews would be more acceptable from an ethical perspective. As is evident from this systematic review, such trials should involve singular interventions since multiple interventions would be difficult to relate to specific impacts. In addition, to identify culprit drugs, such trials have to be large enough to provide information on the contribution of particular drugs or drug classes such as sedatives or antihypertensives on particular elements of the frailty syndrome.

An additional point of attention in designing such trials is the heterogeneity of frailty definitions and frailty assessment scores that measure different aspects of frailty in a non-specific manner. Importantly, a recent review comparing 35 different frailty scores concluded that “research results based on different frailty scores cannot be compared or pooled” [53]. In this regard, recent efforts have been made in trying to find a consensus for functional status across available assessments of physical function or physical frailty [54]. It is also highly likely that such trials would have to be international, involving cooperative consortia with considerable public funding. Clearly, pharmaceutical companies will not be motivated to funding trials on negative outcomes of certain drugs. Furthermore, most of those drugs under suspicion in relation to age-related frailty are generic, further minimizing the level of interest among pharmaceutical companies in funding RCT investigation of the kind.

### Limitations

This systematic review was restricted to MEDLINE entries and to prespecified search terms; thus, relevant literature may have been overlooked. However, the likelihood of missing relevant trials with only one entry, for example exclusively reported in EMBASE, was considered to be low as most trials have multiple citations referring to each other. Unpublished studies were not searched for, e.g., by contacting study investigators or sponsors. The interpretation of results was mainly done by two researchers who may have misinterpreted some findings. Besides, publication bias might be present, i.e., lack of publication of trials with neutral or even negative effects on frailty or frailty components.

## Conclusion

In summary, this review shows that there is virtually no prospective evidence for causal pharmacological effects on frailty with the exception of few trials demonstrating the positive impact of medications on partial aspects of frailty. Dedicated, large prospective trials are urgently needed to identify both the deleterious and/or beneficial effects of drugs on frailty.

## Electronic supplementary material

ESM 1 (DOC 64 kb)

ESM 2 (DOCX 13 kb)

ESM 3 (DOCX 12 kb)

ESM 4 (DOCX 39 kb)

## References

[1] Rodríguez-Laso Á, Mora MAC, Sánchez IG, et al. (2018) State of the art report on the prevention and management of frailty. [Accessed March 17, 2020]. Available from: http://advantageja.eu/images/SoAR-AdvantageJA_Fulltext.pdf

[2] WHO Clinical Consortium on Healthy Ageing, Topic focus: frailty and intrinsic capacity, Report of consortium meeting 1–2 December 2016 in Geneva, Switzerland, [Accessed March 17, 2020]. Available from: https://apps.who.int/iris/bitstream/handle/10665/272437/WHO-FWC-ALC-17.2-eng.pdf

[3] Hoogendijk EO, Afilalo J, Ensrud KE, Kowal P, onder G, Fried LP (2019). Frailty: implications for clinical practice and public health. Lancet..

[4] Kojima G, Liljas AEM, Iliffe S (2019). Frailty syndrome: implications and challenges for health care policy. Risk Manag Healthc Policy.

[5] Palmer K, Villani ER, Vetrano DL (2019). Association of polypharmacy and hyperpolypharmacy with frailty states: a systematic review and meta-analysis. Eur Geriatr Med.

[6] Collard RM, Boter H, Schoevers RA (2012). Prevalence of frailty in community-dwelling older persons: a systematic review. J Am Geriatr Soc.

[7] Vermeiren S, Vella-Azzopardi R, Beckwée D (2016). Frailty and the prediction of negative health outcomes: a meta-analysis. J Am Med Dir Assoc.

[8] Khezrian M, Myint PK, McNeil C (2017). A review of frailty syndrome and its physical, cognitive and emotional domains in the elderly. Geriatrics (Basel).

[9] Dent E, Kowal P, Hoogendijk EO (2016). Frailty measurement in research and clinical practice: a review. Eur J Intern Med.

[10] Mitnitski AB, Mogilner AJ, Rockwood K (2001). Accumulation of deficits as a proxy measure of aging. ScientificWorldJournal..

[11] Gutiérrez-Valencia M, Izquierdo M, Cesari M, Casas-Herrero Á, Inzitari M, Martínez-Velilla N (2018). The relationship between frailty and polypharmacy in older people: a systematic review. Br J Clin Pharmacol.

[12] Muhlack DC, Hoppe LK, Saum KU, Haefeli WE, Brenner H, Schöttker B (2019). Investigation of a possible association of potentially inappropriate medication for older adults and frailty in a prospective cohort study from Germany. Age Ageing.

[13] Maher LM, Hanlon, Hajjar ER (2014). Clinical consequences of polypharmacy in elderly. Expert Opin Drug Saf.

[14] Dent E, Martin FC, Bergman H, Woo J, Romero-Ortuno R, Walston JD (2019). Management of frailty: opportunities, challenges, and future directions. Lancet..

[15] Dent E, Morley JE, Cruz-Jentoft AJ, Arai H, Kritchevsky SB, Guralnik J, Bauer JM, Pahor M, Clark BC, Cesari M, Ruiz J, Sieber CC, Aubertin-Leheudre M, Waters DL, Visvanathan R, Landi F, Villareal DT, Fielding R, Won CW, Theou O, Martin FC, Dong B, Woo J, Flicker L, Ferrucci L, Merchant RA, Cao L, Cederholm T, Ribeiro SML, Rodríguez-Mañas L, Anker SD, Lundy J, Gutiérrez Robledo LM, Bautmans I, Aprahamian I, Schols JMGA, Izquierdo M, Vellas B (2018). International clinical practice guidelines for sarcopenia (ICFSR): screening, diagnosis and management. J Nutr Health Aging.

[16] Palmer K, Marengoni A, Russo P, Mammarella F, onder G (2016). Frailty and drug use. J Frailty Aging.

[17] PRISMA Checklist, [Accessed March 17, 2020]. Available from: http://prisma-statement.org/prismastatement/Checklist.aspx

[18] Paccagnella A, Favaretto A, Oniga F, Barbieri F, Ceresoli G, Torri W, Villa E, Verusio C, Cetto GL, Santo A, de Pangher V, Artioli F, Cacciani GC, Parodi G, Soresi F, Ghi MG, Morabito A, Biason R, Giusto M, Mosconi P, Chiarion Sileni V, GSTVP (Gruppo di Studio Tumori Polmonari del Veneto) (2004). Cisplatin versus carboplatin in combination with mitomycin and vinblastine in advanced non small cell lung cancer. A multicenter, randomized phase III trial. Lung Cancer.

[19] Jadad AR, Moore RA, Carroll D (1996). Assessing the quality of reports of randomized clinical trials: is blinding necessary?. Control Clin Trials.

[20] Buta BJ, Walston JD, Godino JG, Park M, Kalyani RR, Xue QL, Bandeen-Roche K, Varadhan R (2016). Frailty assessment instruments: systematic characterization of the uses and contexts of highly-cited instruments. Ageing Res Rev.

[21] Kenny AM, Kleppinger A, Annis K, Rathier M, Browner B, Judge JO, McGee D (2010). Effects of transdermal testosterone on bone and muscle in older men with low bioavailable testosterone levels, low bone mass, and physical frailty. J Am Geriatr Soc.

[22] Li CM, Chen CY, Li CY, et al. (2010) The effectiveness of a comprehensive geriatric assessment intervention program for frailty in community-dwelling older people: a randomized, controlled trial. Arch Gerontol Geriatr. Suppl 1:S39–42 10.1016/S0167-4943(10)70011-X 20171455

[23] Romera-Liebana L, Orfila F, Segura JM (2018). Effects of a primary care-based multifactorial intervention on physical and cognitive function in frail, elderly individuals: a randomized controlled trial. J Gerontol A Biol Sci Med Sci.

[24] Matchar DB, Duncan PW, Lien CT, Ong MEH, Lee M, Gao F, Sim R, Eom K (2017). Randomized controlled trial of screening, risk modification, and physical therapy to prevent falls among the elderly recently discharged from the emergency department to the community: the steps to avoid falls in the elderly study. Arch Phys Med Rehabil.

[25] van Lieshout MRJ, Bleijenberg N, Schuurmans MJ, de Wit NJ (2018). The effectiveness of a PRoactive multicomponent intervention program on disability in independently living older people: a randomized controlled trial. J Nutr Health Aging.

[26] Setiati S, Anugrahini, Fransiska JE (2018). Combination of alfacalcidol and calcium improved handgrip strength and mobility among Indonesian older women: A randomized controlled trial. Geriatr Gerontol Int.

[27] Wouters H, Scheper J, Koning H, Brouwer C, Twisk JW, van der Meer H, Boersma F, Zuidema SU, Taxis K (2017). Discontinuing inappropriate medication use in nursing home residents: a cluster randomized controlled trial. Ann Intern Med.

[28] Ueda K, Kasao M, Shimamura M, Haruta H, Nitta S, Kaneko M, Uemura Y, Morita H, Komuro I, Shirai T (2016). Impact of oral treatment on physical function in older patients hospitalized for heart failure: a randomized clinical trial. PLoS One.

[29] Aspenberg P, Malouf J, Tarantino U, García-Hernández PA, Corradini C, Overgaard S, Stepan JJ, Borris L, Lespessailles E, Frihagen F, Papavasiliou K, Petto H, Caeiro JR, Marin F (2016). Effects of teriparatide compared with risedronate on recovery after pertrochanteric hip fracture: results of a randomized, active-controlled, double-blind clinical trial at 26 weeks. J Bone Joint Surg Am.

[30] Potter K, Flicker L, Page A, Etherton-Beer C (2016). Deprescribing in frail older people: a randomised controlled trial. PLoS One.

[31] Marek KD, Stetzer F, Ryan PA, Bub LD, Adams SJ, Schlidt A, Lancaster R, O’Brien AM (2013). Nurse care coordination and technology effects on health status of frail older adults via enhanced self-management of medication: randomized clinical trial to test efficacy. Nurs Res.

[32] Burton LA, Sumukadas D, Witham MD, Struthers AD, McMurdo MET (2013). Effect of spironolactone on physical performance in older people with self-reported physical disability. Am J Med.

[33] Beyer I, Bautmans I, Njemini R, Demanet C, Bergmann P, Mets T (2011). Effects on muscle performance of NSAID treatment with piroxicam versus placebo in geriatric patients with acute infection-induced inflammation. A double blind randomized controlled trial. BMC Musculoskelet Disord.

[34] Singh NA, Quine S, Clemson LM, Williams EJ, Williamson DA, Stavrinos TM, Grady JN, Perry TJ, Lloyd BD, Smith EUR, Singh MAF (2012). Effects of high-intensity progressive resistance training and targeted multidisciplinary treatment of frailty on mortality and nursing home admissions after hip fracture: a randomized controlled trial. J Am Med Dir Assoc.

[35] Travison TG, Basaria S, Storer TW (2011). Clinical meaningfulness of the changes in muscle performance and physical function associated with testosterone administration in older men with mobility limitation. J Gerontol A Biol Sci Med Sci.

[36] Atkinson RA, Srinivas-Shankar U, Roberts SA (2010). Effects of testosterone on skeletal muscle architecture in intermediate-frail and frail elderly men. J Gerontol A Biol Sci Med Sci.

[37] White HK, Petrie CD, Landschulz W, MacLean D, Taylor A, Lyles K, Wei JY, Hoffman AR, Salvatori R, Ettinger MP, Morey MC, Blackman MR, Merriam GR, for the Capromorelin Study Group (2009). Effects of an oral growth hormone secretagogue in older adults. J Clin Endocrinol Metab.

[38] Tse W, Frisina PG, Hälbig TD (2008). The effects of withdrawal of dopaminergic medication in nursing home patients with advanced parkinsonism. J Am Med Dir Assoc.

[39] Bent S, Xu L, Lui LY, Nevitt M, Schneider E, Tian G, Guo S, Cummings S (2003). A randomized controlled trial of a Chinese herbal remedy to increase energy, memory, sexual function, and quality of life in elderly adults in Beijing, China. Am J Med.

[40] Liu PY, Wishart SM, Handelsman DJ (2002). A double-blind, placebo-controlled, randomized clinical trial of recombinant human chorionic gonadotropin on muscle strength and physical function and activity in older men with partial age-related androgen deficiency. J Clin Endocrinol Metab.

[41] Hébert R, Robichaud L, Roy PM, Bravo G, Voyer L (2001). Efficacy of a nurse-led multidimensional preventive programme for older people at risk of functional decline. A randomized controlled trial. Age Ageing.

[42] Fiatarone Singh MA, Bernstein MA, Ryan AD (2000). The effect of oral nutritional supplements on habitual dietary quality and quantity in frail elders. J Nutr Health Aging.

[43] McMurdo ME, Millar AM, Daly F (2000). A randomized controlled trial of fall prevention strategies in old peoples' homes. Gerontology..

[44] Coleman EA, Grothaus LC, Sandhu N, Wagner EH (1999). Chronic care clinics: a randomized controlled trial of a new model of primary care for frail older adults. J Am Geriatr Soc.

[45] Moher D, Pham B, Jones A (1998). Does quality of reports of randomised trials affect estimates of intervention efficacy reported in meta-analyses?. Lancet..

[46] Blenkinsopp A, Bond C, Raynor DK (2012). Medication reviews. Br J Clin Pharmacol.

[47] By the 2019 American Geriatrics Society Beers Criteria® Update Expert Panel (2019). American Geriatrics Society 2019 Updated AGS Beers Criteria® for Potentially Inappropriate Medication Use in Older Adults. J Am Geriatr Soc.

[48] Pazan F, Weiss C, Wehling M (2019). The **FORTA** (Fit fOR The Aged) **List** 2018: Third Version of a Validated Clinical Tool for Improved Drug Treatment in Older People. Drugs Aging.

[49] Gallagher P, Ryan C, Byrne S, Kennedy J, O’Mahony D (2008). STOPP (screening tool of older Person's prescriptions) and START (screening tool to alert doctors to right treatment). Consensus validation. Int J Clin Pharmacol Ther.

[50] Cerreta F, European Medicines Agency Geriatric Expert Group (2019) New harmonized considerations on the evaluation instruments for baseline characterization of frailty in the European Union. Br J Clin Pharmacol 10.1111/bcp.14044 PMC7495266 31276597

[51] Alvarez CA, Mortensen EM, Makris UE, Berlowitz DR, Copeland LA, Good CB, Amuan ME, Pugh MJV (2015). Association of skeletal muscle relaxers and antihistamines on mortality, hospitalizations, and emergency department visits in elderly patients: a nationwide retrospective cohort study. BMC Geriatr.

[52] Cho H, Myung J, Suh HS, Kang HY (2018). Antihistamine use and the risk of injurious falls or fracture in elderly patients: a systematic review and meta-analysis. Osteoporos Int.

[53] Aguayo GA, Donneau AF, Vaillant MT (2017). Agreement between 35 published frailty scores in the general population. Am J Epidemiol.

[54] Brefka S, Dallmeier D, Mühlbauer V, von Arnim CAF, Bollig C, onder G, Petrovic M, Schönfeldt-Lecuona C, Seibert M, Torbahn G, Voigt-Radloff S, Haefeli WE, Bauer JM, Denkinger MD, von Arnim CAF, Bauer JM, Bollig C, Brefka S, Dallmeier D, Denkinger MD, Medication and Quality of Life Research Group (2019) A proposal for the retrospective identification and categorization of older people with functional impairments in scientific studies-recommendations of the medication and quality of life in frail older persons (MedQoL) research group. J Am Med Dir Assoc 20:138–146 10.1016/j.jamda.2018.11.008 30638832

