# Correction to: Current evidence on the impact of medication optimization or pharmacological interventions on frailty or aspects of frailty: a systematic review of randomized controlled trials

**DOI:** 10.1007/s00228-021-03166-1

**Published:** 2021-08-07

**Authors:** Farhad Pazan, Mirko Petrovic, Antonio Cherubini, Graziano Onder, Alfonso J. Cruz-Jentoft, Michael Denkinger, Tischa J. M. van der Cammen, Jennifer M. Stevenson, Kinda Ibrahim, Chakravarthi Rajkumar, Marit Stordal Bakken, Jean-Pierre Baeyens, Peter Crome, Thomas Frühwald, Paul Gallaghar, Adalsteinn Guðmundsson, Wilma Knol, Denis O’Mahony, Alberto Pilotto, Elina Rönnemaa, José Antonio Serra-Rexach, George Soulis, Rob J. van Marum, Gijsbertus Ziere, Alpana Mair, Heinrich Burkhardt, Agnieszka Neumann-Podczaska, Katarzyna Wieczorowska-Tobis, Marilia Andreia Fernandes, Heidi Gruner, Dhayana Dallmeier, Jean-Baptiste Beuscart, Nathalie van der Velde, Martin Wehling

**Affiliations:** 1 Clinical Pharmacology Mannheim, Faculty of Medicine Mannheim, Ruprecht-Karls-UniversityofHeidelberg, Theodor-Kutzer-Ufer1-3, 68167 Mannheim, Germany; 2 Section of Geriatrics, Department of Internal Medicine and Paediatrics, Ghent University, Ghent, Belgium; 3 Department of Geriatric Medicine, INRCA Istituto Nazionale Di Ricovero E Cura Per Anziani, Ancona, Italy; 4 Department of Cardiovascular, Endocrine-Metabolic Diseases and Aging, Istituto Superiore Di Sanità, Rome, Italy; 5 Servicio de Geriatría. Hospital Universitario Ramón Y Cajal (IRYCIS), Madrid, Spain; 6 Department of Geriatrics, Alb-Donau, University of Ulm and Geriatric Center Ulm/Alb-DonauAgaplesion Bethesda Hospital, Ulm, Germany; 7 Faculty of Industrial Design Engineering, Delft University of Technology, Delft, Netherlands; 8 Institute of Pharmaceutical Science, King’s College London, London, UK; 9 Faculty of Medicine, Academic Geriatric Medicine, Southampton University, Southampton, UK; 10 Geriatric & Stroke Medicine, Brighton and Sussex Medical School, Brighton, UK; 11 Department of Clinical Science, University of Bergen, Bergen, Norway; 12 AZ Alma, Eeklo, Belgium; 13 University of Luxembourg, Esch-sur-Alzette, Luxembourg; 14 Keele University, Newcastle under Lyme, UK; 15 Department of Acute Geriatrics, Social Medical Center East, Vienna, Austria; 16 School of Medicine, University College Cork, Cambridge, UK; 17 University of Iceland, Geriatrics K4, Landspítali University, Reykjavík, Iceland; 18 Geriatrics, Department of Geriatric Medicine and Expertise Centre Pharmacotherapy in Old Persons, University Medical Center Utrecht, Utrecht University, Utrecht, The Netherlands; 19 DepartmentofMedicine, University College Cork, Cork University Hospital, Cork, Ireland; 20 Department of Geriatric Care, Orthogeriatrics and Rehabilitation, Galliera Hospital, Genoa, Italy; 21 Department of Interdisciplinary Medicine, University of Bari, Bari, Italy; 22 Department of Public Health and Caring Sciences/Geriatrics, Uppsala, Sweden; 23 Geriatric Department, Hospital General Universitario Gregorio Marañón, Madrid, Spain; 24 Instituto de Investigación Sanitaria Gregorio Marañón, Madrid, Spain; 25 Consorcio de Investigación Biomédica en Red: Fragilidad y Envejecimiento Saludable, CIBERFES, Madrid, Spain; 26 Medicine Department, Facultad de Medicina, Universidad Complutense de Madrid, Madrid, Spain; 27 Outpatient Geriatric Assessment Unit, Henry Dunant Hospital Center, Athens, Greece; 28 Department of General Medicine and Geriatric Medicine, Free University, Amsterdam, The Netherlands; 29 Section of Geriatric Medicine, Department of Internal Medicine, Erasmus MC, University Medical Center Rotterdam, Rotterdam, The Netherlands; 30 Department of Geriatric Medicine, Maasstad Hospital, Rotterdam, The Netherlands; 31 Effective Prescribing and Therapeutics, Scottish Government, Edinburgh, EH1 3DG UK; 32 IV. Medical Department, University Hospital Mannheim, Heidelberg University, GeriatricsMannheim, Germany; 33 Geriatric Laboratory Palliative Medicine Department, Poznan University of Medical Sciences, Poznan, Poland; 34 Department of Palliative Medicine, Poznan University of Medical Sciences, Poznan, Poland; 35 Palliative Medicine Unit, University Hospital of Lord’s Transfiguration, Poznan, Poland; 36 Department of Internal Medicine, Hospital Curry Cabral, Centro Hospitalar Universitário de Lisboa Central, Lisbon, Portugal; 37 Internal Medicine Department 7.2, Hospital Curry Cabral - Centro Hospitalar Universitário Lisboa Central, EPE, Lisbon, Portugal; 38 AGAPLESION Bethesda Clinic Ulm, Ulm, Germany; 39 Dept.ofEpidemiology, Boston University School of Public Health, Boston, MA USA; 40 Évaluation Des Technologies de Santé Et Des Pratiques Médicales, CHULille, ULR2694-METRICS, Univ. Lille, 59000 Lille, France; 41 DepartmentofGeriatricMedicine, Free University, Amsterdam, the Netherlands

## Correction to: European Journal of Clinical Pharmacology (2021) 77:1–12 https://doi.org/10.1007/s00228-020–02951-8

The article Current evidence on the impact of medication optimization or pharmacological interventions on frailty or aspects of frailty: a systematic review of randomized controlled trials, written by Farhad Pazan, Mirko Petrovic, Antonio Cherubini, Graziano Onder, Alfonso J. Cruz-Jentoft, Michael Denkinger, Tischa J. M. van der Cammen, Jennifer M. Stevenson, Kinda Ibrahim, Chakravarthi Rajkumar, Marit Stordal Bakken, Jean-Pierre Baeyens, Peter Crome, Thomas Frühwald, Paul Gallaghar, Adalsteinn Guðmundsson, Wilma Knol, Denis O’Mahony, Alberto Pilotto, Elina Rönnemaa, José Antonio Serra-Rexach, George Soulis, Rob J. van Marum, Gijsbertus Ziere, Alpana Mair, Heinrich Burkhardt, Agnieszka Neumann-Podczaska, Katarzyna Wieczorowska-Tobis, Marilia Andreia Fernandes, Heidi Gruner, Dhayana Dallmeier, Jean-Baptiste Beuscart, Nathalie van der Velde and Martin Wehling, was originally published electronically on the publisher’s internet portal on 07 August 2020 without open access. With the author(s)’ decision to opt for Open Choice the copyright of the article changed on 14 May 2021 to © The Author(s) 2021 and the article is forthwith distributed under a Creative Commons Attribution 4.0 International License, which permits use, sharing, adaptation, distribution and reproduction in any medium or format, as long as you give appropriate credit to the original author(s) and the source, provide a link to the Creative Commons licence, and indicate if changes were made. The images or other third party material in this article are included in the article’s Creative Commons licence, unless indicated otherwise in a credit line to the material. If material is not included in the article’s Creative Commons licence and your intended use is not permitted by statutory regulation or exceeds the permitted use, you will need to obtain permission directly from the copyright holder. To view a copy of this licence, visit http://creativecommons.org/licenses/by/4.0.

The Original article has been corrected.

