# Supplementary material for: Current evidence on the impact of medication optimization or pharmacological interventions on frailty or aspects of frailty: a systematic review of randomized controlled trials
Source: Eur J Clin Pharmacol. 2020 Aug 7;77(1):1–12. doi: 10.1007/s00228-020-02951-8 (PMC8197722; doi:10.1007/s00228-020-02951-8)
Supplement: Supplementary file 2 — (DOCX 13 kb) [file 228_2020_2951_MOESM2_ESM.docx]

**PICOs and instructions for systematic review of:**

**Definitions of key terms:**

Frailty

Pre-fraily

Frailty instruments

Aspects of frailty

Pharmacological approaches to prevent/treat frailty

Inappropriate prescribing and medication optimization

Polypharmacy

**Aims:**

To review the most reliable evidence on pharmacological approaches to prevent or treat frailty derived from randomized controlled trials (RCT) which included approaches to tackle polypharmacy and inappropriate drug treatment as well as single drug interventions.

**Patient population (P):**

Geriatric patients

**Intervention/tool (I):**

Medication review/drug treatment optimization or drug treatment of frail older patients

**Comparator (C):**

(n/a)

**Outcome (O):**

Changes in frailty status/aspects of frailty

**Study design (S):**

Randomized controlled trials

**Search strategy:**

(“Frailty”[Mesh] OR “Frail Elderly”[Mesh] OR frail*[Title/Abstract] OR frailty[Title/Abstract] OR prefrailty[Title/Abstract] OR prefrail[Title/Abstract] OR “functional decline”[Title/Abstract] OR physical performance[Title/Abstract] OR sppb[Title/Abstract] OR gait speed[Title/Abstract] OR walking speed[Title/Abstract] OR “Timed up and go test”[Title/Abstract] OR “TUG”[Title/Abstract] OR “grip strength”[Title/Abstract]) AND (“Polypharmacy”[Mesh] OR Polypharmacy[Title/Abstract] OR polytherapy[Title/Abstract] OR polymedication[Title/Abstract] OR “medication appropriateness”[Title/Abstract] OR overprescribing[Title/Abstract] OR multidrug[Title/Abstract] OR “medication*”[Title/Abstract] OR “multiple medications”[Title/Abstract] OR “multiple drug*”[Title/Abstract] OR “beers criteria”[Title/Abstract] OR “STOPP AND START”[Title/Abstract] OR “Potentially Inappropriate Medication List”[Mesh] OR “Potentially Inappropriate Medication”[Title/Abstract] OR “Inappropriate Prescribing”[Mesh] OR “Inappropriate Prescribing”[Title/Abstract] OR “Drug Therapy, Combination”[Mesh] OR “Pharmaceutical Preparations”[Mesh] OR “pharmacotherapy”[TW] OR “pharmacist review”[TW] OR “pharmacist intervention”[TW] OR “pharmacist assessment”[TW] OR “pharmacist management”[TW] OR “pharmacist evaluation”[TW] OR “clinical assessment tool”[Title/Abstract] OR “decision support system”[Title/Abstract])

**Filters:**

("Randomized Controlled Trial" [Publication Type]) AND ("humans"[MeSH Terms])

**Years considered:**

January 1, 1998 - October 14, 2019

("1998/01/01"[PDAT] : "3000/12/31"[PDAT])
