# Supplementary material for: Current evidence on the impact of medication optimization or pharmacological interventions on frailty or aspects of frailty: a systematic review of randomized controlled trials
Source: Eur J Clin Pharmacol. 2020 Aug 7;77(1):1–12. doi: 10.1007/s00228-020-02951-8 (PMC8197722; doi:10.1007/s00228-020-02951-8)
Supplement: Supplementary file 3 — (DOCX 12 kb) [file 228_2020_2951_MOESM3_ESM.docx]

**Search strategy**

Two authors (FP and MW) made a proposal for the search terms which was sent to all EuGMS SIG group members for discussion and amendment. Hence, the following search terms (Medical Subject Headings [MeSH] and keywords) and combinations were developed and used in MEDLINE: (“Frailty”[Mesh] OR “Frail Elderly”[Mesh] OR frail*[Title/Abstract] OR frailty[Title/Abstract] OR prefrailty[Title/Abstract] OR prefrail[Title/Abstract] OR “functional decline”[Title/Abstract] OR physical performance[Title/Abstract] OR sppb[Title/Abstract] OR gait speed[Title/Abstract] OR walking speed[Title/Abstract] OR “Timed up and go test”[Title/Abstract] OR “TUG”[Title/Abstract] OR “grip strength”[Title/Abstract]) **AND** (“Polypharmacy”[Mesh] OR Polypharmacy[Title/Abstract] OR polytherapy[Title/Abstract] OR polymedication[Title/Abstract] OR “medication appropriateness”[Title/Abstract] OR overprescribing[Title/Abstract] OR multidrug[Title/Abstract] OR “medication*”[Title/Abstract] OR “multiple medications”[Title/Abstract] OR “multiple drug*”[Title/Abstract] OR “beers criteria”[Title/Abstract] OR “STOPP AND START”[Title/Abstract] OR “Potentially Inappropriate Medication List”[Mesh] OR “Potentially Inappropriate Medication”[Title/Abstract] OR “Inappropriate Prescribing”[Mesh] OR “Inappropriate Prescribing”[Title/Abstract] OR “Drug Therapy, Combination”[Mesh] OR “Pharmaceutical Preparations”[Mesh] OR “pharmacotherapy”[TW] OR “pharmacist review”[TW] OR “pharmacist intervention”[TW] OR “pharmacist assessment”[TW] OR “pharmacist management”[TW] OR “pharmacist evaluation”[TW] OR “clinical assessment tool”[Title/Abstract] OR “decision support system”[Title/Abstract]) **AND** ("Randomized Controlled Trial" [Publication Type]) **AND** ("1998/01/01"[PDAT] : "3000/12/31"[PDAT]) **AND** ("humans"[MeSH Terms]). We restricted our search to MEDLINE.
