# Supplementary material for: Current evidence on the impact of medication optimization or pharmacological interventions on frailty or aspects of frailty: a systematic review of randomized controlled trials
Source: Eur J Clin Pharmacol. 2020 Aug 7;77(1):1–12. doi: 10.1007/s00228-020-02951-8 (PMC8197722; doi:10.1007/s00228-020-02951-8)
Supplement: Supplementary file 4 — (DOCX 39 kb) [file 228_2020_2951_MOESM4_ESM.docx]

**Supplementary Table 1a** Summary of randomized controlled trials on the impact of drug optimization or pharmacological interventions in frail older patients, which utilized comprehensive frailty score and/or aspects of frailty

| **PMID/Author/Year** | **Type of population** | **Age mean (SD if provided)** | **Number of study participants** | **Women (%)** | **Outcome relating to a** comprehensive **frailty score** | **Outcome(s) relating to partial aspects of frailty** | **Intervention/**  **duration** | **Positive outcome(s) relating to frailty** | **Jadad score^*^** |
| --- | --- | --- | --- | --- | --- | --- | --- | --- | --- |
| 20722847/[Kenny AM et al.](https://www.ncbi.nlm.nih.gov/pubmed/?term=20722847)/2010 | Community-dwelling older men with frailty | 77.1  (7.6) | 131 | 0 | **Fried**  **Frailty Criteria & Short Physical Performance Battery (as part of the physical performance tests)** | Strength (handgrip & leg press), physical performance (Physical Activity Scale for the Elderly (PASE) score, 8 ft walk, Chair rise time, Single leg stance, Supine to Stand, Get up and go), body composition (whole body fat, whole body lean and appendicular skeletal mass) | 5 mg/d testosterone (AndroGel™) vs. placebo for 12–24 months. All participants received calcium (1500 mg/d diet and supplement) and cholecalciferol (1000 IU/d) | No significant change in frailty status but a significant increase in lean mass in the testosterone group was observed. In addition, no differences in strength or physical performance was detected. There were no differences in safety parameters. | 3 |
| 20171455/[Li CM et al.](https://www.ncbi.nlm.nih.gov/pubmed/?term=20171455)/2010 | Community-dwelling older people | 78.8  (8.4) | 310 | 47.7 | **Fried Frailty Criteria** | Barthel index (BI) | Comprehensive geriatric assessment (CGA) and appropriate intervention by medication adjustment, exercise instruction, nutrition support, physical rehabilitation, social worker consultation, and specialty referral. The control group received screening evaluation only/  (follow-up for 6 months) | No signiﬁcant differences were observed between the two groups. But the CGA and the following intervention showed a favourable (non-significant) outcome in frail and prefrail older people based on the frailty status and Barthel index. | 1 |
| 29346524/[Romera-Liebana L et al.](https://www.ncbi.nlm.nih.gov/pubmed/?term=29346524)/2018 | Community-dwelling adults aged older than 65 | 77.3 | 352 | 75.3 | **Short Physical Performance Battery (SPPB)** | Physical dimensions including handgrip strength, Functional Reach Test, Unipodal Station Test and neuropsychological performance as measured by Short and Medium-Term Verbal Memory, Animal Naming Test, evocation of words beginning with one explicit letter, designation of famous people’s names, Verbal designation of images and verbal abstraction of word pairs | Four component intervention: exercise training, intake of high protein  nutritional shakes, memory training, and medication review. Control group received standard care. Both groups were also  given counselling regarding dietary habits, lifestyle recommendations,  and domestic hazards. /  12 weeks | SPPB, handgrip strength, functional reach, neurocognitive battery improved significantly in the intervention group as compared to the control group at 3- and 18-months follow-up | 2 |
| 28202383/[Matchar DB et al.](https://www.ncbi.nlm.nih.gov/pubmed/?term=28202383)/2017 | Older people aged 65 years or over who were recently discharged from the emergency department to the community | 77.8 | 354 | 77.1 | **Short Physical Performance Battery (SPPB)** | - | A tailored program of physical therapy focused on progressive training in strength, balance and gait for 3 months plus screening and follow-up for vision, polypharmacy (participants with polypharmacy were referred to a nurse for medication review and reconciliation), and environmental hazards. The control group received usual care prescribed educational materials on falls prevention/  3 months | The SPPB score in the control group deteriorated significantly more than in the intervention group | 3 |
| 30379302/[van Lieshout MRJ et al.](https://www.ncbi.nlm.nih.gov/pubmed/?term=30379302)/2018 | community-dwelling, 65 years and over | 74  (7.2) | 290 | 55 | - | ADL, physical fitness (handgrip strength),  IADL, walking speed (Timed Up and Go test), functional capacity (six-meter walk test), mobility (Morton Mobility Index), feelings of  depression (Hospital Anxious Depression Scale) and loneliness (Jong Gierveld Loneliness Scale) | Four component intervention: medication review and optimization of medication use, improvement of physical fitness, social skills and nutrition. The control group received standard care/  23 weeks | IADL, walking speed and functional capacity improved significantly after the 12-month follow-up. Right-hand grip strength (physical fitness) improved immediately after the intervention but not after the 12- month follow-up | **3** |
| 29143428/[Setiati S et al.](https://www.ncbi.nlm.nih.gov/pubmed/?term=29143428)/2018 | Women aged 60 years or over registered at a geriatric clinic | Estimated  72 | 95 | 100 | - | Handgrip strength and the Timed‐Up and Go test (TUG) | 0.5 mcg/day alfacalcidol plus 500 mg calcium/day in the intervention group vs. placebo plus 500mg calcium/day in the control group/  90 days | Significant improvement of handgrip strength and the median time for the Timed‐Up and Go test (TUG) in the intervention group as compared to the control group | 4 |
| 29052691/[Wouters H et al.](https://www.ncbi.nlm.nih.gov/pubmed/?term=29052691)/2017 | Nursing home residents | 83.5 | 426 | 67.6 | - | Short form of the Severe Impairment Battery (SIB-S), Mini-Mental State Examination (MMSE), Neuropsychiatric Inventory–Nursing Home Version (NPI-NH) | Single Multidisciplinary  Multistep Medication Review (3MR)/  45 minutes on average.  Standard care in the control group/ 45 minutes | No difference between the intervention and control group | 3 |
| 27959941/[Ueda K et al.](https://www.ncbi.nlm.nih.gov/pubmed/?term=27959941)/2016 | Hospitalized patients for heart failure | 82.4 | 59 | 50.9 | - | Barthel Index (BI), Functional Independence Measure (FIM) and its subscales for analysis of both motor and cognitive function, daily steps counted by pedometers | Continuous intravenous infusion therapy with diuretics (widely used) vs. early switch to oral treatment with diuretics/  mean durations of continuous IV infusion in the OM group and  IV group were 2.6 days and 9.8 days | Barthel index score and number of daily steps were significantly higher in the oral treatment group | 3 |
| 27852903/[Aspenberg P et al.](https://www.ncbi.nlm.nih.gov/pubmed/?term=27852903)/2016 | Patients recovering from pertrochanteric hip fracture | 77  (8) | 224 | 77 | - | Timed Up-and-Go (TUG) test | Teriparatide (20 μg/day) vs. risedronate (35 mg/week) after fixation of a low-trauma pertrochanteric hip fracture/  26 weeks | Patients treated with teriparatide needed significantly less time to complete the TUG at 6 and 12 weeks | 5 |
| 26942907/[Potter K et al.](https://www.ncbi.nlm.nih.gov/pubmed/?term=26942907)/2016 | People aged over 65 years living in residential aged care facilities | 84.3  (6.9) | 95 | 52 | - | Mini Mental Status Examination (MMSE), Modified Barthel Index (MBI) | The intervention group received a deprescribing intervention, the planned cessation of non-beneficial medicines. The control group received standard care/  12 months | No significant differences between the two groups | 3 |
| 23817284/[Marek KD et al.](https://www.ncbi.nlm.nih.gov/pubmed/?term=23817284)/2013 | Persons discharged from Medicare-certified home health care agencies | 79.2 | 414 | 66.2 | - | Mini-Mental Status  Examination (MMSE), Physical Performance Test according to Reuben,  Geriatric Depression Scale | Coordinated care by nurses for two intervention groups who also received either an ‘MD.2 medication-dispensing  Machine’ or a medplanner (simple box with separate compartments for  individual medication times). ‘The control group received no intervention beyond pharmacy screen’. | Those with nurse care coordination and the  medplanner had significantly better outcomes than the control group, but the  addition of the MD.2 to nurse care coordination did not further improve clinical outcomes | 2 |
| 23706520/[Burton LA et al.](https://www.ncbi.nlm.nih.gov/pubmed/?term=23706520)/2013 | Community-dwelling participants aged ≥65 years | 75  (6) | 120 | 45.8 | - | The 6-minute walk test, the Incremental Shuttle Walk Test, the Timed Up and Go test, Functional Limitation Profile (as a measure of physical, psychologic, and social function) | 25 mg spironolactone vs. identical placebo daily for 20 weeks in older participants with self-reported issues with activities of daily living/ 20 weeks | No significant difference between the two groups. | 4 |
| 22208783/[Beyer I et al.](https://www.ncbi.nlm.nih.gov/pubmed/?term=22208783)/2011 | Patients aged ≥ 70 years admitted to an acute geriatric ward | 83.3  (6.9) | 30 | 67.9 | - | Changes in muscle mass, muscle performance (grip strength, fatigue resistance and grip work), mobility as evaluated by the Elderly Mobility Scale, lean body mass | Piroxicam 10 mg + a proton pump inhibitor vs. placebo + proton pump inhibitor daily /10 days | Elderly mobility scale, fatigue resistance and grip work significantly improved with piroxicam but not with placebo. There was no significant difference for other parameters. | 2 |
| 21944168/[Singh NA et al.](https://www.ncbi.nlm.nih.gov/pubmed/?term=21944168)/2012 | Patients admitted to public hospital for surgical repair of hip fracture | 79 | 124 | 69 | - | Basic and instrumental activities of daily living and assistive device utilization | A year of high-intensity weight-lifting exercise and treatment of balance, osteoporosis, nutrition, vitamin D/calcium, depression, cognition, vision, home safety, polypharmacy, hip protectors, self-efficacy, and social support vs. standard care as provided for hip fracture including orthogeriatric care, rehabilitation service, other medical and allied health consultation and physiotherapy/  12 months | Basic ADLs declined less and assistive device use was significantly lower at 12 months in the intervention group compared with controls | 3 |
| 21697501/[Travison TG et al.](https://www.ncbi.nlm.nih.gov/pubmed/?term=21697501)/2011 | Community-dwelling men, aged 65 years and older | 74  (5.4) | 209 | 0 | - | Leg-press strength, chest-press strength, grip strength, leg press power, chest press power, stair-climb, 40-m walk, muscle mass, physical activity, gait speed, lean body mass, appendicular lean soft tissue, self-reported function, and fatigue | 10 g testosterone gel vs. placebo daily/  6 months | Greater improvements in leg-press strength, chest-press strength and power, body composition and loaded stair-climb in the intervention group as compared to the control group and significantly greater proportion of participants receiving testosterone improved their leg-press and chest-press strengths and stair-climbing power more than minimally important difference. No improvement in physical activity, walking speed, self-reported function, and fatigue through the intervention. | 4 |
| 20601412/[Atkinson RA et al.](https://www.ncbi.nlm.nih.gov/pubmed/?term=20601412)/2010 | community-dwelling men aged ≥65 years with one or more of Fried’s frailty criteria | 72.6  (5.7) | 30 | 0 | - | Muscle architecture (muscle thickness, fascicle length and pennation angle) assessed by ultrasound | Transdermal testosterone (50 mg) vs. placebo gel daily/  6 months | Transdermal testosterone therapy resulted in a significant preservation of muscle thickness at 6 months while it declined in the placebo group. No significant effect of therapy on fascicle length or pennation angle. | 3 |
| 19174493/[White HK et al.](https://www.ncbi.nlm.nih.gov/pubmed/?term=19174493)/2009 | Community-dwelling older people | 72.8 | 395 | 59 | - | Body composition and physical performance: total body weight, lean body mass, fat body mass, maximum gait speed, stair climb, 6-min walk, 5 chair rises, habitual gait speed, tandem stand, tandem walk | 12 months of treatment with the orally active GHS capromorelin to 4 dosing groups (10 mg three times/week, 3 mg twice a day, 10 mg each night, and 10 mg twice a day) or placebo/  12 months | At 6 months, body weight increased significantly in participants treated with capromorelin and declined in those treated with placebo. Lean body mass increased and tandem walk ameliorated in the pooled treatment vs. placebo groups. At 12 months, stair climb improved too. | 1 |
| 18992700/[Tse W et al.](https://www.ncbi.nlm.nih.gov/pubmed/?term=18992700)/2008 | Ambulatory nursing home population with parkinsonism | 82  (10.14) | 11 | 36.4 | - | Mini-Mental State Examination (MMSE) | The intervention group underwent levodopa medication withdrawal and the control group continued their treatment with levodopa/  4 weeks | No significant changes were observed between the control and intervention groups in all endpoints. Only 2 of the drug withdrawal patients showed modest improvements in the MMSE | 2 |
| 14698542/[Paccagnella A et al.](https://www.ncbi.nlm.nih.gov/pubmed/14698542)/2004 | Patients  with advanced  or  metastatic non  small cell lung cancer | 59.5 | 153 | 50.3 | - | Activities of daily living AND physical functioning, cognitive functioning, fatigue, appetite loss according to the European Organisation for Research and Treatment of Cancer (EORTC) QoL questionnaires (QLQ-C30+QLQ-LC13) | MVP regimen (Mitomycin-C 8 mg/m2 d1, Vinblastine 4 mg/m2 d 1-8, Cisplatin 100 mg/m2 d1) or MVC regimen (Mitomycin-C 8 mg/m2 d1, Vinblastine 4 mg/m2 d 1-8, Carboplatin 300 mg/m2 d1) every 3 weeks | Significantly less appetite loss in the MVC arm | 1 |
| 14563500/[Bent S et al.](https://www.ncbi.nlm.nih.gov/pubmed/?term=14563500)/2003 | Community-dwelling adults aged 60 or older | 66 | 237 | 63.3 | - | Memory (validated tests of short-term word recall, long-term word recall and picture recall) and physical performance (assessed using ﬁve previously validated tests: 30-s chair-stand, Fatigue and recovery measurement using a 480-s protocol, handgrip strength, A self-paced step test, 6-minute walk, that were modiﬁed slightly by Bent et al.) | Tablets of a Chinese herbal formula (included: Herba Epimedii, Fructus Lycii Chinensis, Cordyceps Sinensis, Herba Cynomorii Songarici, Herba Cistanches, Rhizoma Polygonati, Radix Astragali, Radix Rehmanniae, Glutinosae Conquitae and Radix Morindae Officinalis) or of an identical placebo,  three times a day for 30 days/  30 days | No improvement in physical performance, memory or strength. | 5 |
| 12107212/[Liu PY et al.](https://www.ncbi.nlm.nih.gov/pubmed/?term=12107212)/2002 | Community-dwelling men more than 60 years old | 67.5  (0.8) | 40 | 0 | - | Body weight, lean mass, physical activity (accelerometer and Physical Activity Scale of the Elderly: PASE), physical function (functional tests of static and dynamic balance, functional reach, chair rise, and self-selected and fast gait), muscle mass, muscle strength (Cybex NORM dynamometer) | 3 months of treatment with s.c. recombinant human chorionic gonadotropin (r-hCG) vs. placebo/  3 months | r-hCG significantly increased body weight and lean body mass and reduced fat mass. Other relevant parameters were unchanged. | 3 |
| 11395345/[Hébert R et al.](https://www.ncbi.nlm.nih.gov/pubmed/?term=11395345)/2001 | Community-dwelling older people aged over 75 | 80.3 | 503 | 64.2 | - | Functional Autonomy Measurement System (SMAF), Dupuy’s General Well-being Schedule | Intervention consisted of assessment by a nurse on 12 dimensions including drug treatment and recommendations to participants GPs. Monthly telephone calls were made by the nurse to verify if the recommendations had been implemented/  12 months | No difference between the two groups. | 3 |
| 10828934/[Fiatarone Singh MA et al.](https://www.ncbi.nlm.nih.gov/pubmed/?term=10828934)/2000 | Nursing home residents | 88  (1) | 50 | 31 | - | Anthropometric measurements, body composition analysis (weight, BMI, sum 7 skinfolds, mid-arm muscle area, CT muscle, CT fat, total body water, total body potassium, physical and functional performance tests: muscle strength in hip and knee, gait velocity, habitual physical activity measured by an activity monitor | Supplementation with a multinutrient liquid supplement vs. a non-nutritive placebo drink/  10 weeks | A small but significant gain in weight, increases in fat stores, but no improvement in lean tissue mass through the intervention. No physical performance or functional gains were associated with supplementation. No change in muscle strength. | 1 |
| 10671804/[McMurdo ME et al.](https://www.ncbi.nlm.nih.gov/pubmed/?term=10671804)/2000 | Elderly people living in  residential care | 84  (6.8) | 133 | 81.2 | - | Functional reach, reaction time, timed up-and go (TUG), grip strength, spinal flexibility, and Philadelphia Geriatric Centre Morale Scale and Mini-Mental State Examination (MMSE) | Medication review/falls risk factor assessment/  modification and seated balance exercise training program vs. reminiscence therapy/  6 months | No difference between the two groups. | 2 |
| 10404919/[Coleman EA et al.](https://www.ncbi.nlm.nih.gov/pubmed/?term=10404919)/1999 | Older patients from ambulatory care clinics | 77.3 | 169 | 48.5 | - | Depressive symptoms (CES-D Depression), physical  function: Medical Outcomes Study Short Form (36) Health Survey (MOS SF-36) | Intervention practices  held ‘half-day Chronic Care Clinics’ every 3 to 4  months. These clinics included an extended visit with the  physician and nurse with a special focus on chronic disease management; a pharmacist visit that aimed at a reduction of  polypharmacy and high-risk medications; and a patient self-management/support group. Control practices  received standard care/  24 months | No difference between the two groups. | 2 |

* The Jadad score which is a scale to assess the methodological quality or risk of bias of clinical trials is calculated by using a three-item questionnaire. Drop-outs/withdrawals, randomization, blinding and the quality of latter two items are assessed. The derived score ranges from zero (very poor) to five (rigorous). Jadad AR, Moore RA, Carroll D, et al. Assessing the quality of reports of randomized clinical trials: is blinding necessary? Control Clin Trials. 1996 Feb;17:1-12.

**Supplementary Table 1b** Details regarding medication review/medication optimization (if used)

| **Author/Year** | **Details on medication review/medication optimization** |
| --- | --- |
| [Li CM et al.](https://www.ncbi.nlm.nih.gov/pubmed/?term=20171455)/2010 | Medication adjustment by two board-certified geriatricians |
| [Romera-Liebana L et al.](https://www.ncbi.nlm.nih.gov/pubmed/?term=29346524)/2018 | Reduction of potentially inappropriate medications by a general practitioner who followed the STOPP criteria, with special focus on psychotropic drugs |
| [Matchar DB et al.](https://www.ncbi.nlm.nih.gov/pubmed/?term=28202383)/2017 | A physical therapist referred participants with polypharmacy to a nurse for medication review and reconciliation |
| [Van Lieshout MRJ et al.](https://www.ncbi.nlm.nih.gov/pubmed/?term=30379302)/2018 | ‘The Prescribing Optimization Method (POM) for those with at least four different drugs was used. Besides, participants who were using 1–3 different drugs were able to join voluntarily. Questions regarding (side) effects, interaction of drugs and the feasibility of daily drug intake and drug use were asked in a structured interview. The interview was performed at the pharmacy and led by a pharmacist who consulted the general practitioner of the patient when it turned out that adaptations regarding the medications were needed. The POM required approximately 15 to 45 minutes.’ (see van Lieshout MRJ et al.) |
| [Wouters H et al.](https://www.ncbi.nlm.nih.gov/pubmed/?term=29052691)/2017 | Single Multidisciplinary Multistep Medication Review (3MR) by physicians and pharmacists using the STOPP/START and Beers criteria. |
| [Potter K et al.](https://www.ncbi.nlm.nih.gov/pubmed/?term=26942907)/2016 | A list of potential target medicines for deprescribing and a deprescribing algorithm were used by a general practitioner, a geriatrician or a clinical pharmacologist to stop the use of non-beneficial medicines |
| [Marek KD et al.](https://www.ncbi.nlm.nih.gov/pubmed/?term=23817284)/2013 | Coordinated care by nurses for two intervention groups who also received either an ‘MD.2 medication-dispensing  Machine’ or a medplanner (simple box with separate compartments for  individual medication times) |
| [Singh NA et al.](https://www.ncbi.nlm.nih.gov/pubmed/?term=21944168)/2012 | Addressing polypharmacy through medication review by a geriatrician, consultation with General Practitioner, education of patient/caregiver and completion of medical record card from pharmacy |
| [Hébert R et al.](https://www.ncbi.nlm.nih.gov/pubmed/?term=11395345)/2001 | Intervention consisted of assessment by a nurse on drug treatment and recommendations to participants General Practitioners. Monthly telephone calls were made by the nurse to verify if the recommendations had been implemented |
| [McMurdo ME et al.](https://www.ncbi.nlm.nih.gov/pubmed/?term=10671804)/2000 | Drugs that may have contributed to hypotension were reviewed, and dose reduction, discontinuation, or substitution was recommended. Besides, a routine medication review was performed after which the clinical assessor contacted a general practitioner and made recommendations regarding dose reduction, therapeutic substitution, or discontinuation. The general practitioner changed the drug treatment if necessary |
| [Coleman EA et al.](https://www.ncbi.nlm.nih.gov/pubmed/?term=10404919)/1999 | A 15-minutes pharmacist visit at the primary care examination room that aimed at a reduction of  polypharmacy and high-risk medications associated with functional decline. The list of high-risk medication was developed from the existing literature and after discussions with national experts |

STOPP: Screening Tool of Older Person's Prescriptions; START: Screening Tool to Alert doctors to Right Treatment
